# Supplementary material for: Fabrication of Nitrogen-Doped Carbon@Magnesium Silicate Composite by One-Step Hydrothermal Method and Its High-Efficiency Adsorption of As(V) and Tetracycline
Source: Materials (Basel). 2023 Jul 29;16(15):5338. doi: 10.3390/ma16155338 (PMC10420030; doi:10.3390/ma16155338)
Supplement: Supplementary file 1 [file materials-16-05338-s001.zip › materials-2482621-supplementary.pdf]

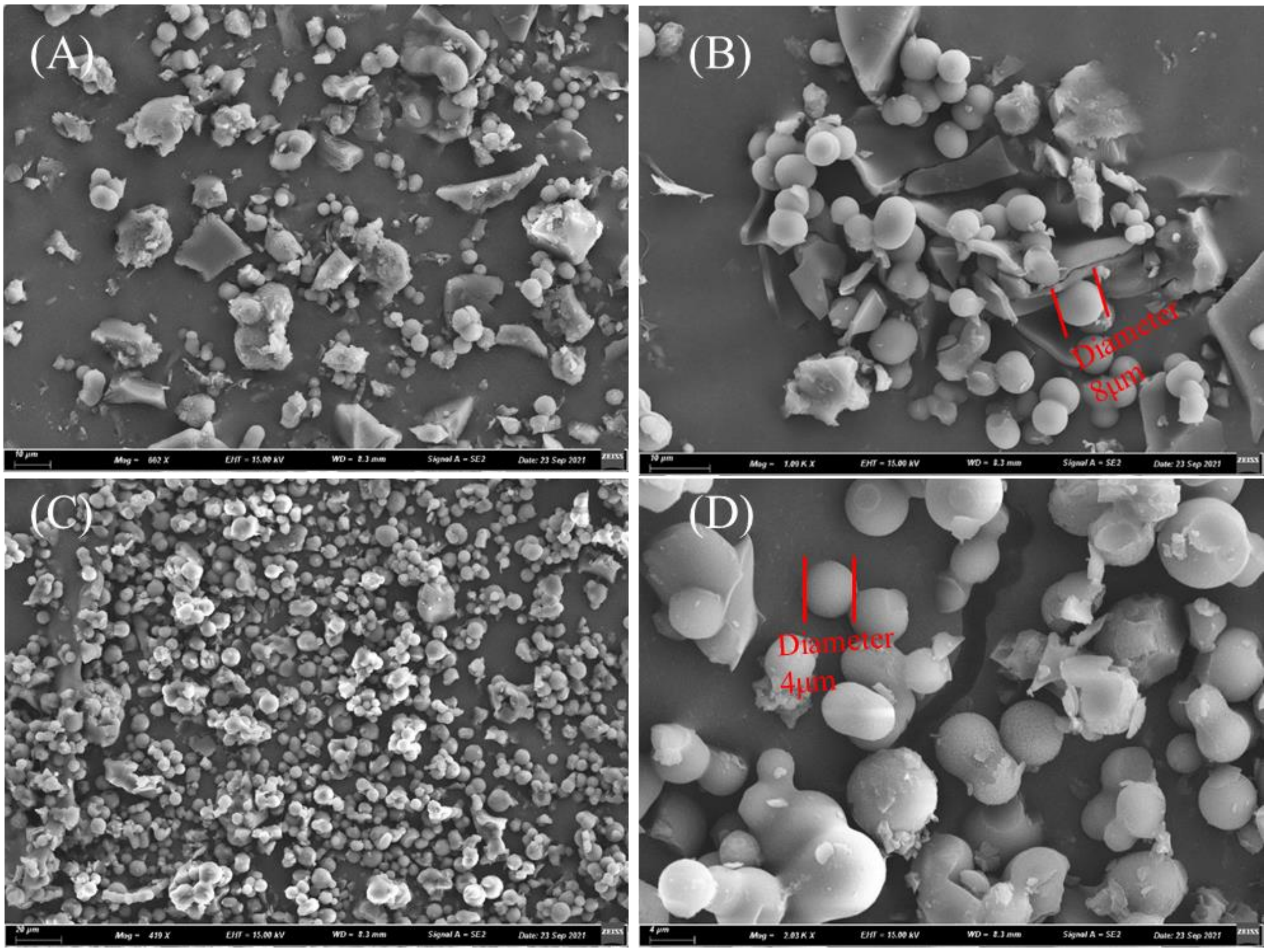

Fig. S1 SEM images of fabricated pure carbon for (A, B) without and (C, D) with ethanol added

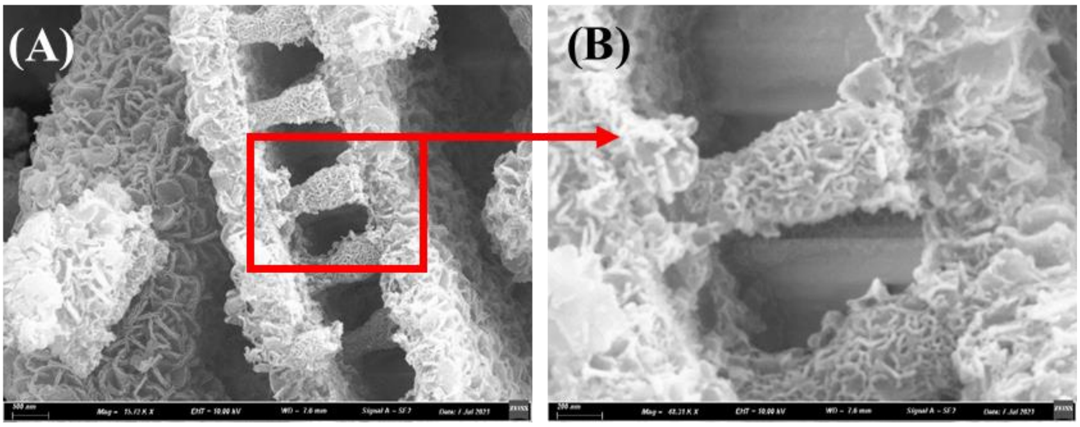

Fig. S2 SEM images of CMS internal structure: cavity structure (A) and nanocolumn array (B)

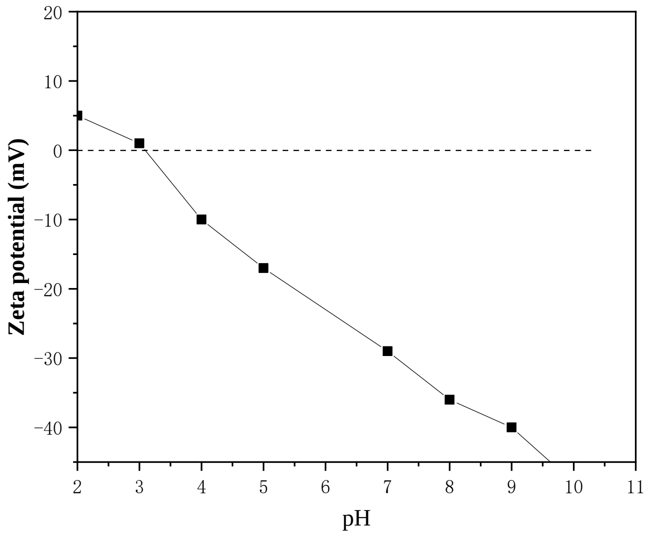

Fig. S3 Zeta potential of CMS at different pH

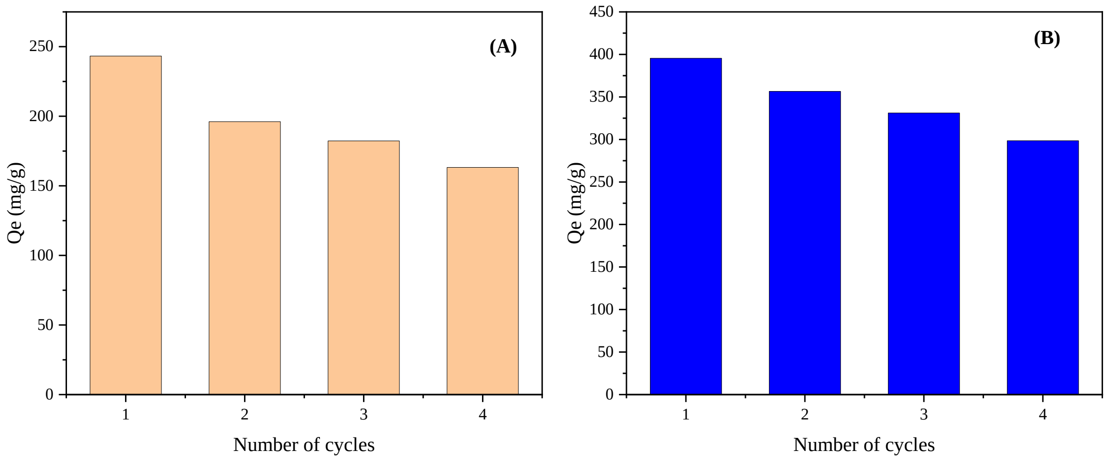

Fig. S4 The number of recycles of CMS for the As(V) (A) and TC (B) adsorption (C_0=200mg/L, m=0.02g, V=70mL)

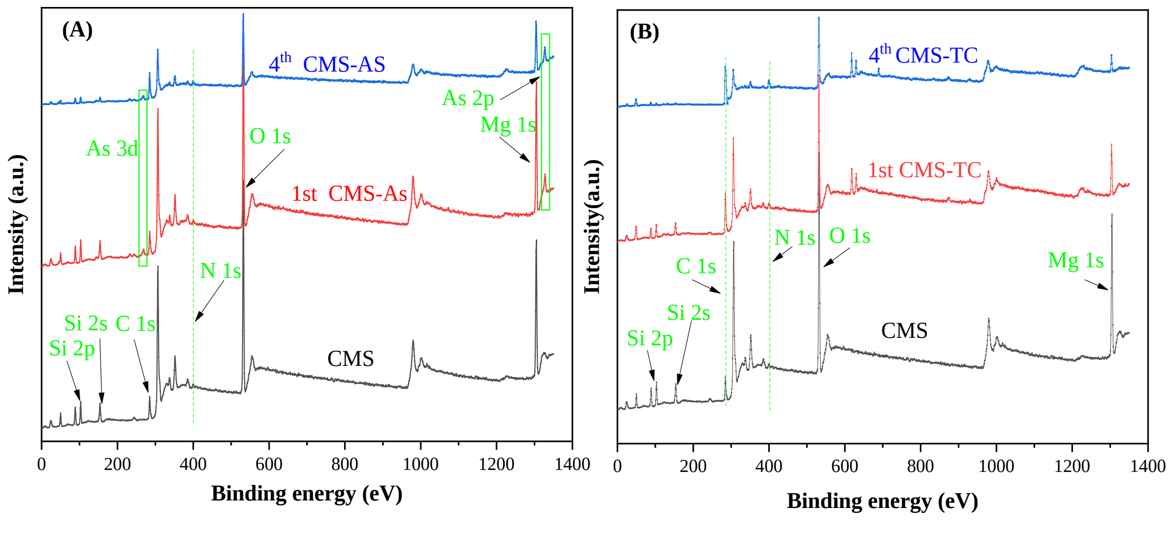

Fig. S5 XPS survey of CMS before and after the 1st and 4th As(V)(A), TC(B) adsorption.

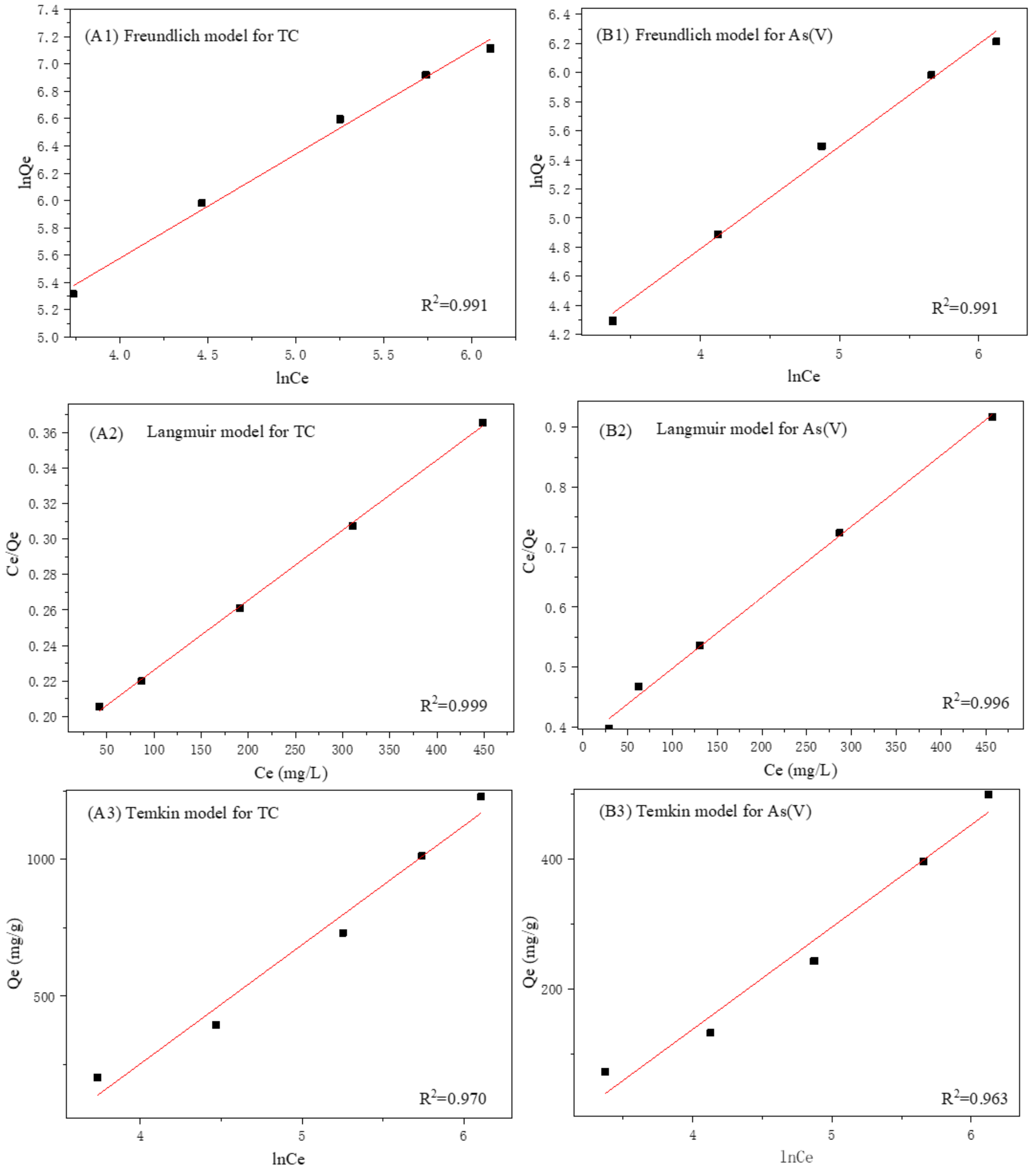

Fig. S6 Adsorption isotherms of TC and As(V) on CMS at 298K, (A1, B1) Freundlich model,(A2, B2) Langmuir model, (A3, B3) Temkin model

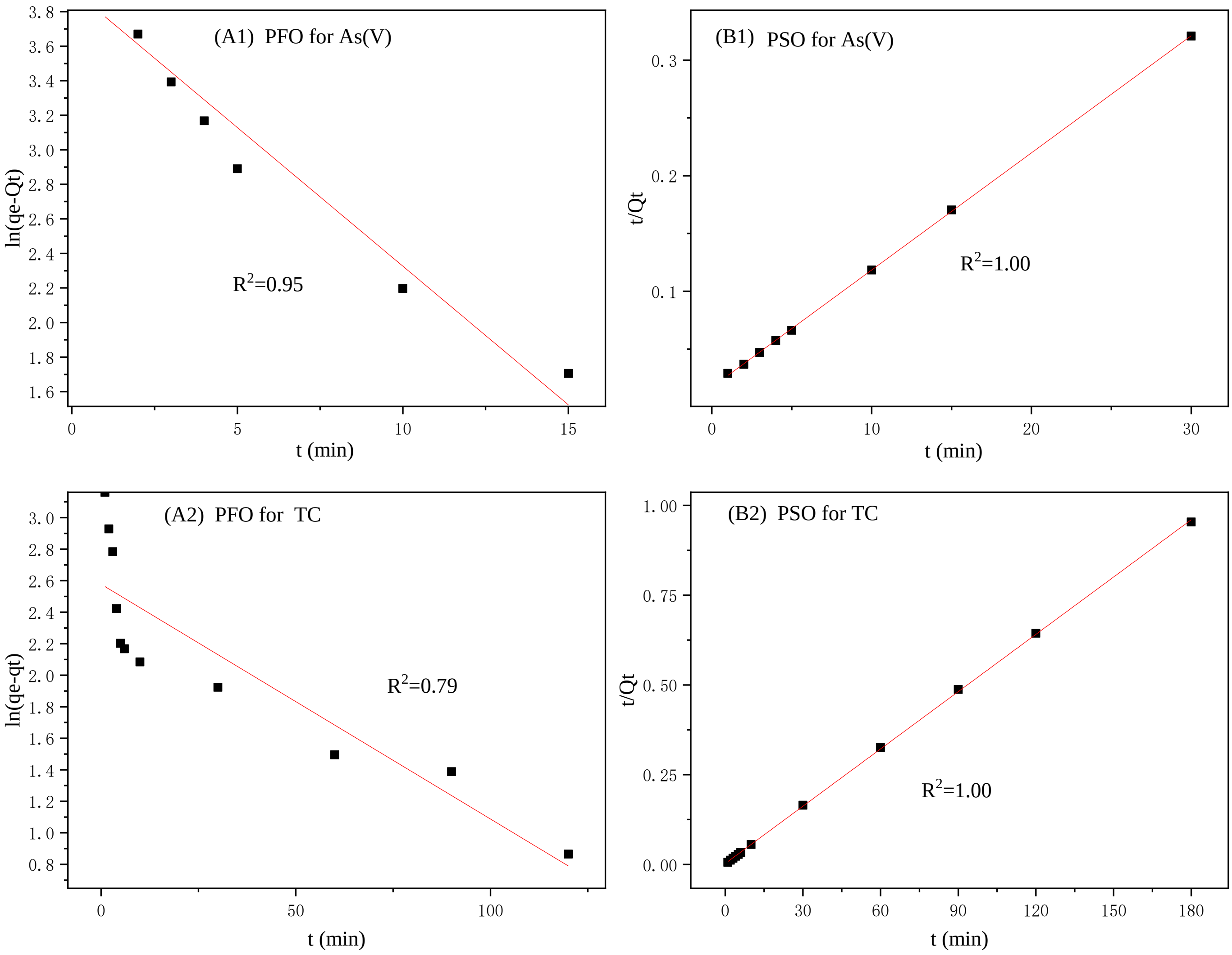

Fig. S7 Kinetics linearly fitting of (A1, A2) PFO and (B1, B2) PSO for As(V) and TC, respectively

Table S1. Concentration of Mg, C and N in different samples.

Species	Concentration (%)				
	CMS	1st CMS-As(V)	4th CMS-As(V)	1st CMS-TC	4th CMS-TC
Mg	18.83	13.89	13.81	8.32	3.22
C	15.35	13.23	11.25	35.45	61.32
N	10.78	4.34	1.63	5.57	3.16
As	/	2.63	2.64	/	/

Texts

Text S1 Isotherms model for the adsorption

The Langmuir equation could be expressed as equation (S1)

$$\frac{C_e}{q_e} = \frac{1}{q_m \times k_L} + \frac{C_e}{q_m} \qquad \text{(S1)}$$

The Freundlich isotherm could be given as equation (S2)

$$\ln q_e = lnk_F + \frac{1}{n} lnC_e \qquad \text{(S2)}$$

The Temkin isotherm could be given as equation (S3)

$$q_e = \frac{RT}{b} lnk_T + \frac{RT}{b} lnC_e \qquad \text{(S3)}$$

where q_m (mg/g) is the theoretical maximal adsorption capacity, k_L (L/mg) is equilibrium adsorption free energy function, k_F [(mg/g)(mg/L)$^{1/n}$] and n represent adsorption ability and adsorption intensity constant. b(= -ΔH, kJ/mol) represents adsorption heat, k_T (L/mg) is Temkin model constant, R (8.314kJ/mol) is the universal gas constant and T (298K) is absolute temperature.

Text S2 Kinetics model for the adsorption

The pseudo-first-order kinetic model is described as equation (S4):

$$\ln(q_e - q_t) = \ln q_e - k_1 t \qquad \text{(S4)}$$

The pseudo-second-order kinetic model is described as equation (S5):

$$\frac{t}{q_t} = \frac{1}{k_2 q_e^2} + \frac{t}{q_e} \quad \text{(S5)}$$

where k_1 and k_2 represent the pseudo-first- and pseudo-second-order rate constants, q_e and q_t (mg g^{-1}) denote the adsorption of As(V) and TC at equilibrium and time t (min)

Text S3 Intra-particle diffusion and Boyd model for the adsorption

The intra-particle diffusion kinetic model is described as equation (S6):

$$Qt = k_i \ t^{1/2} + C \quad \text{(S6)}$$

where k_i is a constant related to intra-particle diffusion rate and C is a constant.

The Boyd kinetic model is described as equation (S7):

$$B_t = -\ln(1- q_t/q_e)-0.4977 \quad \text{(S7)}$$

where q_t and q_e (mg/g) are the amounts of As(V), TC adsorbed on the adsorbent at time t and equilibrium time, respectively.
